# Clinical Characteristics and Management of Late Urinary Symptom Flare Following Stereotactic Body Radiation Therapy for Prostate Cancer

**DOI:** 10.3389/fonc.2014.00122

**Published:** 2014-05-26

**Authors:** Jennifer A. Woo, Leonard N. Chen, Aditi Bhagat, Eric K. Oermann, Joy S. Kim, Rudy Moures, Thomas Yung, Siyuan Lei, Brian T. Collins, Deepak Kumar, Simeng Suy, Anatoly Dritschilo, John H. Lynch, Sean P. Collins

**Affiliations:** ^1^Department of Radiation Medicine, Georgetown University Hospital, Washington, DC, USA; ^2^Cancer Research Laboratory, Department of Biology, University of the District of Columbia, Washington, DC, USA; ^3^Department of Urology, Georgetown University Hospital, Washington, DC, USA

**Keywords:** prostate cancer, SBRT, CyberKnife, EPIC, AUA symptom score, genitourinary toxicity, late urinary symptom flare, bother

## Abstract

**Purpose:** Stereotactic body radiation therapy (SBRT) is increasingly utilized as primary treatment for clinically localized prostate cancer. While acute post-SBRT urinary symptoms are well recognized, the late genitourinary toxicity of SBRT has not been fully described. Here, we characterize the clinical features of late urinary symptom flare and recommend conservative symptom management approaches that may alleviate the associated bother.

**Methods:** Between February 2008 and August 2011, 216 men with clinically localized prostate cancer were treated definitively with SBRT at Georgetown University Hospital. Treatment was delivered using the CyberKnife with doses of 35–36.25 Gy in five fractions. The prevalence of each of five Common Terminology Criteria for Adverse Events (CTCAE) graded urinary toxicities was assessed at each follow-up visit. Medication usage was documented at each visit. Patient-reported urinary symptoms were assessed using the American Urological Association (AUA) symptom score and the Expanded Prostate Cancer Index Composite (EPIC)-26 at 1, 3, 6, 9, 12, 18, and 24 months. Late urinary symptom flare was defined as an increase in the AUA symptom score of ≥5 points above baseline with a degree of severity in the moderate to severe range (AUA symptom score ≥15). The relationship between the occurrence of flare and pre-treatment characteristics were examined.

**Results:** For all patients, the AUA symptom score spiked transiently at 1 month post-SBRT. Of the 216 patients, 29 (13.4%) experienced a second transient increase in the AUA symptom score that met the criteria for late urinary symptom flare. Among flare patients, the median age was 66 years compared to 70 for those without flare (*p* = 0.007). In patients who experienced flare, CTCAE urinary toxicities including dysuria, frequency/urgency, and retention peaked at 9–18 months, and alpha-antagonist utilization increased at 1 month post-treatment, rose sharply at 12 months post-treatment, and peaked at 18 months (85%) before decreasing at 24 months. The EPIC urinary summary score of flare patients declined transiently at 1 month and experienced a second, more protracted decline between 6 and 18 months before returning to near baseline at 2-year post-SBRT. Statistically and clinically significant increases in patient-reported frequency, weak stream, and dysuria were seen at 12 months post-SBRT. Among flare patients, 42.9% felt that urination was a moderate to big problem at 12 months following SBRT.

**Conclusion:** In this study, we characterize late urinary symptom flare following SBRT. Late urinary symptom flare is a constellation of symptoms including urinary frequency/urgency, weak stream, and dysuria that transiently occurs 6–18 months post-SBRT. Provision of appropriate anticipatory counseling and the maintenance of prophylactic alpha-antagonists may limit the bother associated with this syndrome.

## Introduction

Due to unavoidable dose to the bladder neck and prostatic urethra, genitourinary (GU) toxicities are common following prostate cancer radiotherapy (1, 2). After external beam radiation therapy, incidence of late GU toxicity (≥grade 2) ranges from 10 to 30% (3–5). Previous studies have recognized older age (6), higher radiation dose (5), and prior transurethral resection of the prostate (TURP) (7) as risk factors for late GU toxicity following external beam radiotherapy. GU toxicities are rarely an isolated finding, occurring more commonly as a complex of lower urinary tract symptoms (LUTS) (8). Recent data suggest that many of these toxicities may resolve with time (9) and analysis of actuarial incidence may over-estimate their clinical significance (10).

Patient responses to validated questionnaires may better define the longitudinal pattern of GU toxicity following radiotherapy compared with physician-reported data (11). Late urinary symptom flare was first described when urinary symptom questionnaires were administered to patients following prostate brachytherapy (12–14) and later confirmed by others (15, 16). Younger age at time of implantation was the only pre-treatment characteristic consistently associated with an increased risk of flare.

A case of late urinary symptom flare following stereotactic body radiation therapy (SBRT) was first reported by Suy et al. (17). It occurred 1-year post-SBRT and the patient eventually underwent a transurethral resection of prostate tissue (TURP). Microscopic evaluation of the TURP specimen suggested an inflammatory process (17). Subsequently, we described a series of patients experiencing post-SBRT late urinary toxicity, which resolved with conservative treatment such as alpha-blockers and/or brief steroid tapers (8). Endoscopic evaluation of these patients revealed bladder neck/urethral hyperemia suggestive of cystourethritis.

While acute GU toxicity post-SBRT is well characterized (8, 18–24), the late GU toxicity of SBRT has not been fully described. Here, we characterize the clinical features of late urinary symptom flare following SBRT, and recommend conservative symptom management approaches that may alleviate bother and minimize unnecessary interventions such as cystoscopy.

## Materials and Methods

### Patient selection

Patients eligible for study inclusion had histologically confirmed, clinically localized adenocarcinoma of the prostate treated per our institutional protocol. Patients eligible for inclusion in this study had a prostate-specific antigen (PSA) <40 ng/ml, clinical stage T1c–T2c, and a Gleason score of ≤8. Exclusion criteria included clinically involved lymph nodes on imaging, distant metastases on bone scan; prior pelvic radiotherapy and/or prior radical prostate surgery. Institutional IRB approval was obtained for retrospective review of data that was prospectively collected in our institutional database.

### Treatment planning and delivery

Stereotactic body radiation therapy treatment planning and delivery were conducted as previously described (25, 26). Briefly, gold fiducials were placed into the prostate. Fused CT and MR images were used for treatment planning. The clinical target volume (CTV) included the prostate and the proximal seminal vesicles. The planning target volume (PTV) equaled the CTV expanded 3 mm posteriorly and 5 mm in all other dimensions. The prescription dose was 35–36.25 Gy to the PTV delivered in five fractions of 7–7.25 Gy over 1–2 weeks (27). The prescription isodose line was limited to ≥75%. The bladder and membranous urethra were contoured and evaluated with dose–volume histogram analysis during treatment planning using Multiplan (Accuray Inc., Sunnyvale, CA, USA) inverse treatment planning. Critical structure dose constraints were as previously described (26). To minimize the risk of local recurrence, no attempt was made to limit the dose to the prostatic urethra. Target position was verified during treatment using paired, orthogonal X-ray images (25, 28).

### Follow-up and statistical analysis

Prostate-specific antigen levels were obtained before treatment, 1 month after the completion of SBRT, and during routine follow-up visits every 3 months for the first year and every 6 months for the second year of follow-up. All medications, including alpha-antagonists, corticosteroids, non-steroidal antiinflammatory drugs (NSAIDs), and urethral analgesics were documented at each visit. Toxicity was assessed prospectively at follow-up visits using the National Cancer Institute (NCI) Common Terminology Criteria for Adverse Events (CTCAE) version 3.0. Patient-reported outcomes were assessed pre-treatment and at follow-up visits using the American Urological Association (AUA) symptom score (29) and the Expanded Prostate Cancer Index Composite (EPIC) short form (30).

Student’s *t*-test was used to assess differences in ongoing PSA levels. Wilcoxon signed rank test and Mann–Whitney *U* test were used to analyze the quality of life scores in comparison to baseline. Sample medians and ranges were used to describe PSA levels. A clinically significant urinary flare was prospectively defined as an AUA symptom score ≥15 with an increase of ≥5 points above baseline (15, 16). Based upon published results, a benign PSA bounce was defined as a PSA rise of 0.2 ng/ml or more above its previous nadir with a subsequent decline to that nadir or lower (31). To statistically compare changes between time points, the levels of responses were assigned a score and the significance of the mean changes in the scores was assessed by paired *t-*test. EPIC scores for the urinary domain and its individual questions range from 0 to 100 with lower values representing worsening urinary symptoms. The minimally important difference (MID) in EPIC score was defined as a change of one-half standard deviation (SD) from the baseline (32).

The impact of baseline patient characteristics on the incidence of late urinary symptom flare was evaluated by univariate and multivariate analyses. Univariate analysis of variance (ANOVA) was used to detect significant relationship between patient characteristics and urinary symptom flare. In multivariate analysis, stepwise ordinal logistic regression modeling was used to determine independent factors predicting late urinary symptom flare. The baseline patient characteristics that were included as variables in the univariate and multivariate analyses included age, race, D’Amico Risk Group, Gleason score, T-Stage, prostate volume, PSA, testosterone level, baseline AUA symptom score, alpha-antagonist usage, baseline EPIC urinary summary score, partner status, work status, Charlson comorbidity index, prior procedure for benign prostatic hyperplasia (BPH), and pre-treatment androgen deprivation therapy (ADT). All tests were two-tailed, and a *p*-value <0.05 was considered significant. IBM^®^ SPSS version 21 and MedCalc^®^ version 12.6.1.0 were used to perform the statistical analyses.

## Results

From August 2008 to August 2011, 216 patients with clinically localized prostate adenocarcinoma were treated per our institutional SBRT monotherapy protocol. The patients were followed for a minimum of 24 months following SBRT. The median patient age was 69 years (48–90 years) (Table 1). The percentage of patients self-identified as white was 56.5 and 37.5% as black. Comorbidities were common. The median prostate volume was 38 cc (11.6–138.7 cc) and 8.8% had prior procedures for BPH. There were 38.4% low-risk patients, 51.4% patients were intermediate-risk, and 10.2% patients were high-risk. Twenty-nine patients (13.4%) also received ADT. A dose of 36.25 Gy delivered in five 7.25 Gy fractions was used for 87.5% of patients.

**Table 1:** **Patient demographics and characteristics**.

		Total (*N* = 216)	No flare (*N* = 187)	Flare (*N* = 29)
Age (years)		Median 69 (48–90) years/old	Median 70 (49–90) years/old	Median 66 (48–77) years/old
Race	White	122 (56.5%)	109 (58.3%)	13 (44.8%)
	Black	81 (37.5%)	65 (34.8%)	16 (55.2%)
	Other	13 (6.0%)	13 (7.0%)	0 (0%)
Partner status	Partnered	162 (75.0%)	138 (73.8%)	24 (82.8%)
	Not partnered	54 (25%)	49 (26.2%)	5 (17.2%)
Work status	Work	99 (45.8%)	87 (46.5%)	12 (41.4%)
	Not employed	117 (54.2%)	100 (53.5%)	17 (58.6%)
Charlson comorbidity index (CCI)	CCI = 0	146 (64.6%)	127 (67.9%)	19 (65.5%)
	CCI = 1	51 (23.6%)	44 (23.5%)	7 (24.1%)
	CCI ≥ 2	19 (8.8%)	16 (8.6%)	3 (10.3%)
T-stage	T1c	160 (74.1%)	139 (74.3%)	21 (72.4%)
	T2a	29 (13.4%)	25 (13.4%)	4 (13.8%)
	T2b	20 (9.3%)	17 (9.1%)	3 (10.3%)
	T2c	7 (3.2%)	6 (3.2%)	1 (3.4%)
Gleason	6	97 (44.9%)	83 (44.4%)	14 (48.3%)
	7	109 (50.5%)	94 (50.3%)	15 (51.7%)
	8	10 (4.6%)	10 (5.3%)	0 (0%)
D’Amico risk groups	Low	83 (38.4%)	71 (38.0%)	12 (41.4%)
	Intermediate	111 (51.4%)	95 (50.8%)	16 (55.2%)
	High	22 (10.2%)	21 (11.2%)	1 (3.4%)
Prostate volume (cc)		Median 38 (11.6–138.7)	Median 37.6 (11.6–108)	Median 39.5 (13.8–138.7)
PSA (ng/ml)		Median 5.8 (0.2–32.5)	Median 5.8 (0.2–32.5)	Median 5.7 (1.3–17.5)
Testosterone (ng/dL)		Median 302.5 (20–1149)	285.5 (20–1149)	342 (40–692)
AUA score		Median 7.5 (0–33)	Median 8 (0–33)	Median 7 (1–17)
EPIC urinary domain		Median 88.9 (40.8–97.2)	Median 88.9 (40.8–97.2)	Median 94.4 (60.2–97.2)
α_1A_ Antagonist usage		62 (28.7%)	54 (28.9%)	8 (27.6%)
Procedures for BPH		19 (8.8%)	18 (9.6%)	1 (3.5%)
ADT	With ADT	29 (13.4%)	26 (13.9%)	3 (10.3%)
SBRT dose (Gy)	35	27 (12.5%)	23 (12.3%)	4 (13.8%)
	36.25	189 (87.5%)	164 (87.7%)	25 (86.2%)

*PSA, prostate-specific antigen; AUA, American Urological Association symptom score; EPIC, expanded prostate cancer index composite; BPH, benign prostate hyperplasia; ADT, androgen deprivation therapy; SBRT, stereotactic body radiation therapy*.

**Figure 1:**
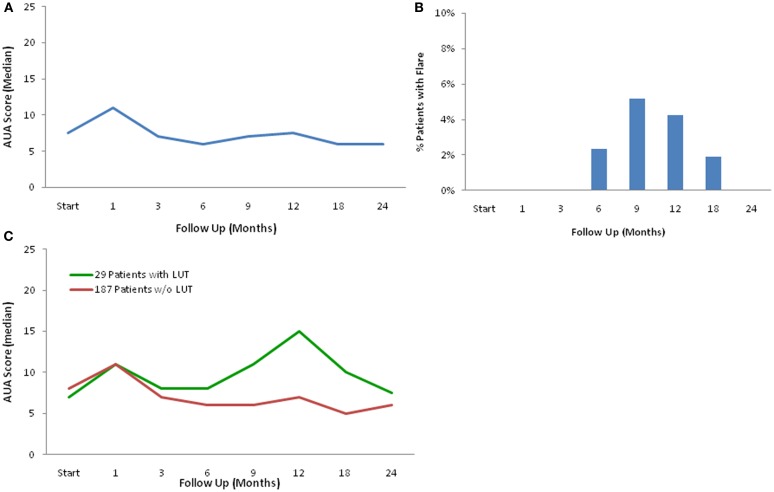
**Late urinary symptom flare**. **(A)** AUA symptom score at baseline and following SBRT for prostate cancer. **(B)** Percentage of patients with urinary symptom flare at each follow-up. **(C)** AUA symptom score in patients with and without late urinary symptom flare. Thresholds for clinically significant changes in scores (1/2 standard deviation above and below the baseline). AUA scores range from 0 to 35 with higher values representing worsening urinary symptoms.

The mean AUA symptom score increased transiently at 1 month post-SBRT and returned to near baseline by 3 months post-SBRT (Figure 1A). This acute increase was both statistically (*p* < 0.0001) and clinically significant (MID = 2.99). A second late protracted increase occurred between 9 and 18 months (Figure 1A). Transient late urinary symptom flare (occurring ≥6 months after completing treatment) occurred in 13.4% of the patients (Figure 1B). The median flare magnitude was 13 and the median time to flare was 9 months (range, 6–18 months) (Figure 1C). The mean AUA score reduced to below baseline at 2 years post-SBRT. Among flare patients, the median age was 66 years compared to 70 for those without flare (*p* = 0.007) (Table 2). No other baseline patient characteristics were significantly associated with flare following SBRT.

**Table 2:** **Impact of baseline patient characteristics on the incidence of late urinary symptom flare 2 years post-SBRT**.

Factors	*p*-Value
Age	0.007*†
Race	0.505
D’Amico risk groups	0.381
Gleason score (6, 7, ≥8)	0.453
T-stage (T1c, palpable)	0.865
Prostate volume	0.820
PSA	0.299
Testosterone level	0.111
Initial AUA	0.080
α1A antagonist usage	0.975
Initial EPIC urinary domain	0.166
Partner status	0.812
Work status	0.599
Charlson comorbidity index	0.794
Procedure for BPH	0.212
Androgen deprivation therapy	0.282

*Significant in multivariate analysis; ^†^significant in univariate analysis.

*PSA, prostate-specific antigen; AUA, American Urological Association urinary symptom score; EPIC, expanded prostate cancer index composite; BPH, benign prostate hypertrophy*.

A PSA bounce of 0.2 ng/ml occurred in 30.1% of the study group of 216 patients. A total of 9/29 patients (31.0%) of the flare patients experienced a PSA bounce compared with 56/187 (29.9%) of the non-flare patients who experienced a bounce. Of the nine patients who experienced both a late urinary symptom flare and benign PSA bounce, two experienced the bounce and flare on the same follow-up evaluation. In three patients, the PSA bounce occurred before the flare, and in four patients the bounce occurred after the symptom flare.

**Figure 2:**
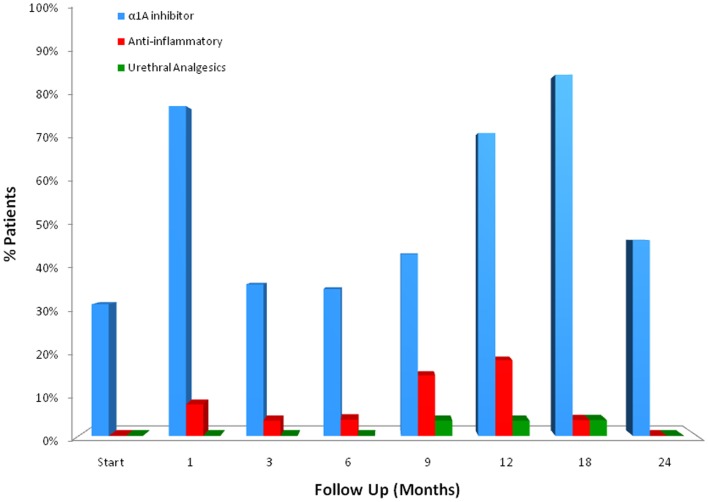
**Medication utilization for urinary symptom relief by flare patients at baseline and following SBRT for prostate cancer**.

For patients who experienced late urinary symptom flare, the prevalence of each of five CTCAE-graded urinary toxicities (hematuria, dysuria, incontinence, frequency, urgency, and retention) during the first 2 years of follow-up is illustrated in Table 3. Dysuria, urinary frequency/urgency, and retention were the most commonly experienced late toxicities. At baseline and each follow-up, usage of medication for urinary symptoms was assessed (Table 4; Figure 2). At baseline, 31% of patients reported using alpha-antagonists; this increased at 1 month post-treatment, rose sharply at 12 months post-treatment, and peaked at 18 months (85.2%) before decreasing at 24 months. Similarly, anti-inflammatory and urethral analgesic use peaked at 12 and 18 months, respectively.

**Table 3:** **Prevalence of CTCAE-graded urinary toxicities in flare patients following SBRT for prostate cancer**.

Toxicity	Follow-up (months)	1	3	6	9	12	18	24
	
	Grade							
Hematuria	0	92.6%	100.0%	96.2%	85.7%	93.1%	89.7%	96.4%
	1	7.4%	0.0%	0.0%	10.7%	6.9%	10.3%	3.6%
	2	0.0%	0.0%	0.0%	0.0%	0.0%	0.0%	0.0%
	3	0.0%	0.0%	3.8%	3.6%	0.0%	0.0%	0.0%
Dysuria	0	74.1%	85.2%	80.8%	75.0%	79.3%	82.8%	96.4%
	1	25.9%	14.8%	19.2%	25.0%	20.7%	13.8%	3.6%
	2	0.0%	0.0%	0.0%	0.0%	0.0%	3.4%	0.0%
Incontinence	0	77.8%	81.5%	76.9%	57.1%	72.4%	62.1%	82.1%
	1	22.2%	18.5%	23.1%	39.3%	27.6%	37.9%	14.3%
	2	0.0%	0.0%	0.0%	3.6%	0.0%	0.0%	3.6%
Urinary frequency/urgency	0	14.3%	44.4%	34.6%	25.0%	24.1%	41.4%	57.1%
	1	75.0%	51.9%	61.5%	60.7%	58.6%	48.3%	42.9%
	2	10.7%	3.7%	3.8%	14.3%	17.2%	10.3%	0.0%
Retention	0	37.0%	51.9%	42.3%	46.4%	41.4%	17.2%	46.4%
	1	7.4%	22.2%	30.8%	17.9%	3.4%	17.2%	17.9%
	2	55.6%	25.9%	26.9%	35.7%	55.2%	65.5%	35.7%

**Table 4:** **Percent of flare patients utilized medications for urinary symptom relief at baseline and following SBRT for prostate cancer**.

	Start	1	3	6	9	12	18	24
α_1A_ Inhibitor (%)	31.03	77.78	35.71	34.62	42.86	71.43	85.19	46.15
Anti-inflammatory (%)	0.00	7.41	3.57	3.85	14.29	17.86	3.70	0.00
Urethral analgesics (%)	0.00	0.00	0.00	0.00	3.57	3.57	3.70	0.00

**Figure 3:**
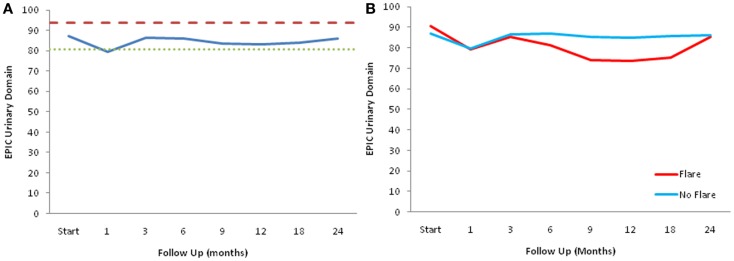
**EPIC urinary summary domain scores**. **(A)** EPIC urinary summary domain scores at baseline and following SBRT for prostate cancer. **(B)** EPIC urinary summary domain scores in patients with and without late urinary symptom flare. Thresholds for clinically significant changes in scores (1/2 standard deviation above and below the baseline) are marked with dashed lines. EPIC scores range from 0 to 100 with higher values representing a more favorable health-related QOL.

**Figure 4:**
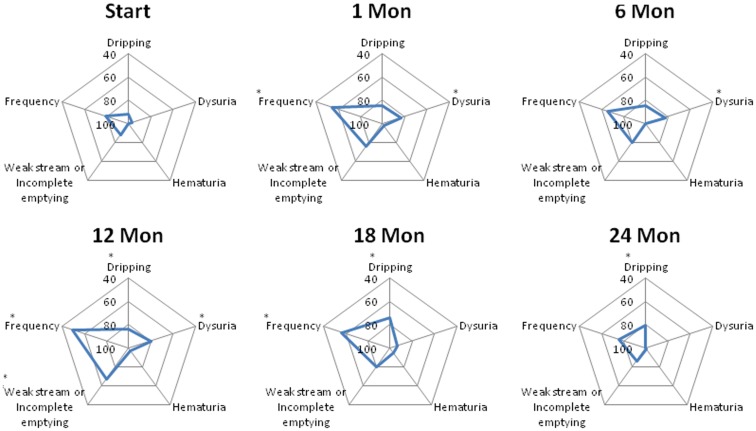
**Spider plots of individual EPIC urinary symptom scores at baseline and following SBRT for prostate cancer**. Dripping-question 4A of the EPIC-26; dysuria-question 4B of the EPIC-26; hematuria-question 4C of the EPIC-26; weak stream or incomplete emptying-question 4D of the EPIC-26; and frequency-question 4E of the EPIC-26. EPIC scores range from 0 to 100 with higher values representing a more favorable health-related QOL. Changes in scores that are both statistically and clinically significant are marked with an asterisk (*).

The declines in the EPIC urinary scores paralleled the increases in the AUA symptom scores. The EPIC urinary summary score declined transiently at 1 month post-SBRT and returned to near baseline by 3 months post-SBRT (Figure 3A). This acute decline was both statistically (*p* < 0.0001) and clinically significant (MID = 6.48). A second late, protracted decline occurred between 9 and 18 months (Figure 3A). Transient late declines in the EPIC urinary summary domain were occurred almost exclusively in patients who experienced late urinary symptom flare (Figure 3B). Statistically and clinically significant increases in patient-reported frequency, weak stream, and dysuria were seen in flare patients at 12 months post-SBRT (Figure 4). The EPIC urinary summary score returned to near baseline by 2 years post-SBRT (Figures 3A,B and 4).

**Figure 5:**
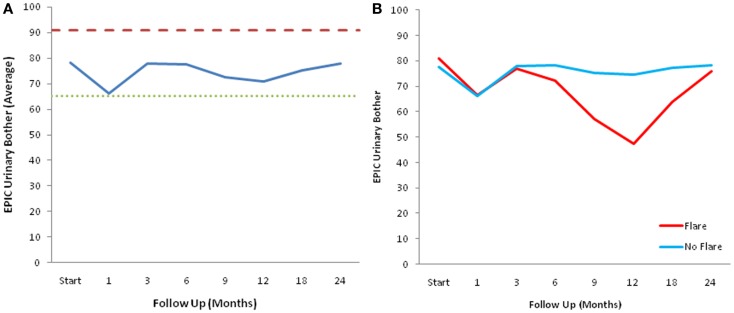
**EPIC urinary bother scores**. **(A)** EPIC urinary bother scores at baseline and following SBRT for prostate cancer. **(B)** EPIC urinary bother scores in patients with and without late urinary symptom flare. Thresholds for clinically significant changes in scores (1/2 standard deviation above and below the baseline) are marked with dashed lines. EPIC scores range from 0 to 100 with higher values representing a more favorable health-related QOL.

At baseline, 9.3% of the patients felt that urination was a moderate to big problem (Table 5). The mean EPIC urinary bother score was 78.2 at baseline (Figure 5A). Urinary bother increased following treatment with the mean score decreasing to 66.4 at 1 month post-treatment (*p* < 0.0001) (Figure 5A). However, only 17.5% of patients felt that urination was a moderate to big problem at 1 month following treatment (Table 5). Although urinary bother declined quickly, a second late increase in urinary bother was observed with the mean urinary bother score decreasing to 70.8 at 12 months (*p* = 0.009) (Figure 5A). Transient late declines in the EPIC urinary bother score were more common in patients who experienced late urinary symptom flare (Figure 5B). At 12 months post-treatment, 42.9% of the flare patients felt that urination was a moderate to big problem (Table 5). By 2 years following SBRT, urinary bother returned to near baseline in both flare and non-flare patients (Figure 5B).

**Table 5:** **Urinary bother in patients with and without late urinary symptom flare following SBRT for prostate cancer (patient-reported responses to question 5 of the EPIC-26.)**.

	Start	1 Month	3 Months	6 Months	9 Months	12 Months	18 Months	24 Months
**ALL PATIENTS**								
*N*	214	211	208	198	197	196	182	196
No problem (%)	46.3	24.6	40.9	42.9	40.1	35.7	44.5	45.4
Very small–small problem (%)	44.4	57.8	50.5	48.0	44.2	49.5	41.8	45.9
Moderate–big problem (%)	9.3	17.5	8.7	9.1	15.7	14.8	13.7	8.7
*p*-Value		<0.0001*	0.9201	0.7601	0.0777	0.0086*	0.4353	0.7329
**PATIENTS WITHOUT FLARE**								
*N*	185	184	180	172	169	168	155	170
No problem (%)	43.8	24.5	42.2	44.8	43.2	38.1	46.5	45.9
Very small–small problem (%)	47.6	58.2	48.9	46.5	43.2	51.8	41.3	45.9
Moderate–big problem (%)	8.6	17.4	8.9	8.7	13.6	10.1	12.3	8.2
*p*-Value		<0.0001*	0.7298	0.9282	0.5778	0.3185	0.6496	0.3608
**PATIENTS WITH FLARE**								
*N*	29	27	28	26	28	28	27	26
No problem (%)	62.1	25.9	32.1	30.8	21.4	21.4	33.3	42.3
Very small–small problem (%)	24.1	55.6	60.7	57.7	50.0	35.7	44.4	46.2
Moderate–big problem (%)	13.8	18.5	7.1	11.5	28.6	42.9	22.2	11.5
*p*-Value		0.0483*	0.5614	0.4307	0.0056*	0.0008*	0.0737	0.3028

*Changes in survey scores that are both statistically and clinically significant.

## Discussion

While conventionally fractionated intensity-modulated radiotherapy (IMRT) and brachytherapy remain the most commonly used radiation therapy modalities for clinically localized prostate cancer, SBRT is increasingly utilized as primary treatment for low to intermediate-risk disease. Data from several single-institution series (8, 20, 22, 33), multi-institutional phase I study (21), and a multi-institutional registry (23, 24) suggest SBRT offers high rates of biochemical control and low rates of grade 3 and higher toxicities, comparable with conventionally fractionated IMRT and brachytherapy. The maturation of these data is reflected in ASTRO’s recent statement that SBRT can be “considered an appropriate alternative for select patients with low to intermediate-risk disease” (ASTRO Model Policies, 2012, https://www.astro.org/Practice-Management/Reimbursement/Model-Policies.aspx). As utilization of SBRT for prostate cancer will likely increase in coming years, it is imperative to understand its impact on patients’ quality of life.

Some studies have suggested an increased incidence of late GU toxicities following hypofractionated radiation therapy when compared to conventionally fractionated treatment (34–36). Our previous study showed a 30% incidence of late grade 2 GU toxicities in the first 2 years following prostate SBRT (8). However, the majority of these patients experienced resolution of their GU toxicities with time. Reporting toxicities as actuarial incidence might overstate the significance of such problems (10, 37). In our opinion, prevalence better reflects the impact of a given toxicity on a patient’s quality of life, and should be the standard approach for reporting toxicities.

Our analysis of the impact of baseline patient characteristics on the incidence of late urinary symptom flare revealed that young age was the only factor associated with an increased risk of developing flare. The association with age has been previously been seen with late urinary symptom flare following brachytherapy (14). To date, no mechanistic rationale has been determined. Surprisingly, pre-treatment urinary function was not significantly associated with flare in this study. Nor could we establish a relationship between the severity of acute urinary symptoms and the incidence of late urinary symptom flare (data not shown) (38).

Endoscopic evaluation of these patients suggests that these symptoms may be caused by radiation-induced cystourethritis. An inflammatory etiology is supported by late urinary symptom flare’s delayed occurrence and rapid symptomatic response to oral steroids, not unlike post-radiation pneumonitis (39, 40). Radiation-induced inflammation is known to be dependent on radiation fractionation and the volume of the target organ irradiated (41, 42). Future studies should evaluate the impact of treatment factors (such as bladder neck/urethral dose) on the incidence of late urinary symptom flare (43). Interestingly, there was no temporal relationship between late urinary symptom flare and benign PSA bounces, suggesting a non-inflammatory nature for post-SBRT PSA bounces (14).

Similar to BPH-associated LUTS, patients with late urinary symptom flare present with obstructive and irritative voiding symptoms. Alpha-blockers have been shown to improve urinary flow and acute urinary symptoms following prostate radiotherapy (44, 45). In this series, most flare patients responded to alpha-antagonists. For those that did not respond adequately, we initiated a short oral corticosteroid taper (dexamethasome 4 mg daily for 1 week followed by 2 mg for 1 week, then discontinue) to reduce inflammation. With these conservative medical approaches, the majority of patients with urinary symptom flare recover to near baseline without the need for invasive procedures. Only a few patients required urethral anesthetics for persistent dysuria. Over the course of longer follow-up, we have not observed flare recurrences beyond 2 years since treatment (data not shown).

Prophylactic alpha-blockers have been shown to reduce urinary morbidity following prostate brachytherapy (46, 47). To maximize patient comfort, it is currently our institutional policy to initiate prophylactic alpha-antagonist use prior to initiating treatment. Patients are encouraged to continue alpha-antagonist usage until they return to their baseline urinary status and to reinitiate alpha-antagonists with any worsening of their urinary function.

We analyzed quality of life questionnaires to determine the degree of bother experienced by patients with late urinary symptom flare. Our results suggest that late urinary flare was highly bothersome to a significant percentage of the patients who experienced it. It is likely that these patients, not anticipating the onset of late urinary symptoms following a transient period of acute symptoms and a symptom-free interval, were particularly sensitive to perceived changes in their urinary status as they progressed further from the completion of SBRT (48). Clinicians should anticipate the possibility of late urinary symptoms and communicate to patients that a late transient flare may occur; this will better prepare patients for the onset of symptoms and potentially mitigate the perceived severity of the flare (48).

## Conclusion

Our data provide compelling evidence for a clinical syndrome of transient late-onset urinary symptoms associated with SBRT for localized prostate cancer. The majority of flares occurs at 9–18 months post-SBRT and resolve by 24 months after treatment. Currently, it is difficult to predict which patients are at the highest risk of late urinary symptom flare. Provision of appropriate anticipatory counseling for all patients prior to treatment and utilization of alpha-antagonists may limit the need for invasive interventions.

## Conflict of Interest Statement

Dr. Sean Collins and Dr. Brian Collins are consultants for Accuray, Inc. The other co-authors declare that the research was conducted in the absence of any commercial or financial relationships that could be construed as a potential conflict of interest.

## References

[1] Bekelman JE Mitra N Efstathiou J Liao K Sunderland R Yeboa DN Outcomes after intensity-modulated versus conformal radiotherapy in older men with nonmetastatic prostate cancer. Int J Radiat Oncol Biol Phys (2011) 81:e325–34 10.1016/j.ijrobp.2011.02.006 21498008 PMC4265571

[2] Michalski JM Yan Y Watkins-Bruner D Bosch WR Winter K Galvin JM Preliminary toxicity analysis of 3-dimensional conformal radiation therapy versus intensity modulated radiation therapy on the high-dose arm of the Radiation Therapy Oncology Group 0126 prostate cancer trial. Int J Radiat Oncol Biol Phys (2013) 87:932–8 10.1016/j.ijrobp.2013.07.041 24113055 PMC3840044

[3] Peeters ST Heemsbergen WD Koper PC Van Putten WL Slot A Dielwart MF Dose-response in radiotherapy for localized prostate cancer: results of the Dutch multicenter randomized phase III trial comparing 68 Gy of radiotherapy with 78 Gy. J Clin Oncol (2006) 24:1990–6 10.1200/JCO.2005.05.2530 16648499

[4] Kuban DA Tucker SL Dong L Starkschall G Huang EH Cheung MR Long-term results of the M. D. Anderson randomized dose-escalation trial for prostate cancer. Int J Radiat Oncol Biol Phys (2008) 70:67–74 10.1016/j.ijrobp.2007.06.054 17765406

[5] Zelefsky MJ Levin EJ Hunt M Yamada Y Shippy AM Jackson A Incidence of late rectal and urinary toxicities after three-dimensional conformal radiotherapy and intensity-modulated radiotherapy for localized prostate cancer. Int J Radiat Oncol Biol Phys (2008) 70:1124–9 10.1016/j.ijrobp.2007.11.044 18313526

[6] Ahmed AA Egleston B Alcantara P Li L Pollack A Horwitz EM A novel method for predicting late genitourinary toxicity after prostate radiation therapy and the need for age-based risk-adapted dose constraints. Int J Radiat Oncol Biol Phys (2013) 86:709–15 10.1016/j.ijrobp.2013.03.020 23664324 PMC3860375

[7] Devisetty K Zorn KC Katz MH Jani AB Liauw SL External beam radiation therapy after transurethral resection of the prostate: a report on acute and late genitourinary toxicity. Int J Radiat Oncol Biol Phys (2010) 77:1060–5 10.1016/j.ijrobp.2009.06.078 20045267

[8] Chen LN Suy S Uhm S Oermann EK Ju AW Chen V Stereotactic Body Radiation Therapy (SBRT) for clinically localized prostate cancer: the Georgetown University experience. Radiat Oncol (2013) 8:58 10.1186/1748-717X-8-58 23497695 PMC3610192

[9] Schmid MP Potter R Bombosch V Sljivic S Kirisits C Dorr W Late gastrointestinal and urogenital side-effects after radiotherapy – incidence and prevalence. Subgroup-analysis within the prospective Austrian-German phase II multicenter trial for localized prostate cancer. Radiother Oncol (2012) 104:114–8 10.1016/j.radonc.2012.05.007 22727264

[10] Peters LJ Withers HR Brown BW Complicating issues in complication reporting. Int J Radiat Oncol Biol Phys (1995) 31:1349–51 10.1016/0360-3016(95)00041-V 7755796

[11] Sonn GA Sadetsky N Presti JC Litwin MS Differing perceptions of quality of life in patients with prostate cancer and their doctors. J Urol (2013) 189:S59–65 10.1016/j.juro.2012.11.032 23234635

[12] Gelblum DY Potters L Ashley R Waldbaum R Wang XH Leibel S Urinary morbidity following ultrasound-guided transperineal prostate seed implantation. Int J Radiat Oncol Biol Phys (1999) 45:59–67 10.1016/S0360-3016(99)00176-5 10477007

[13] Merrick GS Butler WM Lief JH Dorsey AT Temporal resolution of urinary morbidity following prostate brachytherapy. Int J Radiat Oncol Biol Phys (2000) 47:121–8 10.1016/S0360-3016(99)00525-8 10758313

[14] Cesaretti JA Stone NN Stock RG Urinary symptom flare following I-125 prostate brachytherapy. Int J Radiat Oncol Biol Phys (2003) 56:1085–92 10.1016/S0360-3016(03)00210-4 12829146

[15] Crook J Fleshner N Roberts C Pond G Long-term urinary sequelae following 125iodine prostate brachytherapy. J Urol (2008) 179:141–145; discussion 146 10.1016/j.juro.2007.08.136 17997424

[16] Keyes M Miller S Moravan V Pickles T Liu M Spadinger I Urinary symptom flare in 712 125I prostate brachytherapy patients: long-term follow-up. Int J Radiat Oncol Biol Phys (2009) 75:649–55 10.1016/j.ijrobp.2008.11.043 19211199

[17] Suy S Oermann E Hanscom H Lei S Vahdat S Yu X Histopathologic effects of hypofractionated robotic radiation therapy on malignant and benign prostate tissue. Technol Cancer Res Treat (2010) 9:583–7 10.7785/tcrt.2012.500168 21070080

[18] Litwin MS Gore JL Kwan L Brandeis JM Lee SP Withers HR Quality of life after surgery, external beam irradiation, or brachytherapy for early-stage prostate cancer. Cancer (2007) 109:2239–47 10.1002/cncr.22676 17455209

[19] Freeman DE King CR Stereotactic body radiotherapy for low-risk prostate cancer: five-year outcomes. Radiat Oncol (2011) 6:3 10.1186/1748-717X-6-3 21219625 PMC3022740

[20] King CR Brooks JD Gill H Presti JC Jr Long-term outcomes from a prospective trial of stereotactic body radiotherapy for low-risk prostate cancer. Int J Radiat Oncol Biol Phys (2011) 82:877–82 10.1016/j.ijrobp.2010.11.054 21300474

[21] McBride SM Wong DS Dombrowski JJ Harkins B Tapella P Hanscom HN Hypofractionated stereotactic body radiotherapy in low-risk prostate adenocarcinoma: preliminary results of a multi-institutional phase 1 feasibility trial. Cancer (2012) 118:3681–90 10.1002/cncr.26699 22170628

[22] Katz AJ Santoro M Diblasio F Ashley R Stereotactic body radiotherapy for localized prostate cancer: disease control and quality of life at 6 years. Radiat Oncol (2013) 8:118 10.1186/1748-717X-8-118 23668632 PMC3674983

[23] King CR Collins S Fuller D Wang PC Kupelian P Steinberg M Health-related quality of life after stereotactic body radiation therapy for localized prostate cancer: results from a multi-institutional consortium of prospective trials. Int J Radiat Oncol Biol Phys (2013) 87:939–45 10.1016/j.ijrobp.2013.08.019 24119836

[24] King CR Freeman D Kaplan I Fuller D Bolzicco G Collins S Stereotactic body radiotherapy for localized prostate cancer: pooled analysis from a multi-institutional consortium of prospective phase II trials. Radiother Oncol (2013) 109:217–21 10.1016/j.radonc.2013.08.030 24060175

[25] Lei S Piel N Oermann EK Chen V Ju AW Dahal KN Six-dimensional correction of intra-fractional prostate motion with cyberknife stereotactic body radiation therapy. Front Oncol (2011) 1:48 10.3389/fonc.2011.00048 22655248 PMC3356099

[26] Oermann EK Suy S Hanscom HN Kim JS Lei S Yu X Low incidence of new biochemical and clinical hypogonadism following hypofractionated stereotactic body radiation therapy (SBRT) monotherapy for low- to intermediate-risk prostate cancer. J Hematol Oncol (2011) 4:12 10.1186/1756-8722-4-12 21439088 PMC3083385

[27] Katz AJ Santoro M Ashley R Diblasio F Stereotactic body radiation therapy for low- and low-intermediate-risk prostate cancer: is there a dose effect? Front Oncol (2011) 1:49 10.3389/fonc.2011.00049 22655249 PMC3356012

[28] Xie Y Djajaputra D King CR Hossain S Ma L Xing L Intrafractional motion of the prostate during hypofractionated radiotherapy. Int J Radiat Oncol Biol Phys (2008) 72:236–46 10.1016/j.ijrobp.2008.04.051 18722274 PMC2725181

[29] Barry MJ Fowler FJ Jr O’Leary MP Bruskewitz RC Holtgrewe HL Mebust WK The American Urological Association symptom index for benign prostatic hyperplasia. The Measurement Committee of the American Urological Association. J Urol (1992) 148:1549–1557; discussion 1564 1279218 10.1016/s0022-5347(17)36966-5

[30] Wei JT Dunn RL Litwin MS Sandler HM Sanda MG Development and validation of the expanded prostate cancer index composite (EPIC) for comprehensive assessment of health-related quality of life in men with prostate cancer. Urology (2000) 56:899–905 10.1016/S0090-4295(00)00858-X 11113727

[31] Ciezki JP Reddy CA Garcia J Angermeier K Ulchaker J Mahadevan A PSA kinetics after prostate brachytherapy: PSA bounce phenomenon and its implications for PSA doubling time. Int J Radiat Oncol Biol Phys (2006) 64:512–7 10.1016/j.ijrobp.2005.07.960 16213667

[32] Norman GR Sloan JA Wyrwich KW Interpretation of changes in health-related quality of life: the remarkable universality of half a standard deviation. Med Care (2003) 41:582–92 10.1097/00005650-200305000-00007 12719681

[33] Friedland JL Freeman DE Masterson-Mcgary ME Spellberg DM Stereotactic body radiotherapy: an emerging treatment approach for localized prostate cancer. Technol Cancer Res Treat (2009) 8:387–92 19754215 10.1177/153303460900800509

[34] Arcangeli G Fowler J Gomellini S Arcangeli S Saracino B Petrongari MG Acute and late toxicity in a randomized trial of conventional versus hypofractionated three-dimensional conformal radiotherapy for prostate cancer. Int J Radiat Oncol Biol Phys (2011) 79:1013–21 10.1016/j.ijrobp.2009.12.045 20447774

[35] Pollack A Walker G Horwitz EM Price R Feigenberg S Konski AA Randomized trial of hypofractionated external-beam radiotherapy for prostate cancer. J Clin Oncol (2013) 31:3860–8 10.1200/JCO.2013.51.1972 24101042 PMC3805927

[36] Yu JB Cramer LD Herrin J Soulos PR Potosky AL Gross CP Stereotactic body radiation therapy versus intensity-modulated radiation therapy for prostate cancer: comparison of toxicity. J Clin Oncol (2014) 32:1195–201 10.1200/JCO.2013.53.8652 24616315 PMC3986382

[37] Ghadjar P Jackson A Spratt DE Oh JH Munck af Rosenschold P Kollmeier M Patterns and predictors of amelioration of genitourinary toxicity after high-dose intensity-modulated radiation therapy for localized prostate cancer: implications for defining postradiotherapy urinary toxicity. Eur Urol (2013) 64:931–8 10.1016/j.eururo.2013.02.001 23522772 PMC4786022

[38] Dorr W Hendry JH Consequential late effects in normal tissues. Radiother Oncol (2001) 61:223–31 10.1016/S0167-8140(01)00429-7 11730991

[39] Movsas B Raffin TA Epstein AH Link CJ Jr Pulmonary radiation injury. Chest (1997) 111:1061–76 10.1378/chest.111.4.1061 9106589

[40] Graves PR Siddiqui F Anscher MS Movsas B Radiation pulmonary toxicity: from mechanisms to management. Semin Radiat Oncol (2010) 20:201–7 10.1016/j.semradonc.2010.01.010 20685583

[41] Mehta V Radiation pneumonitis and pulmonary fibrosis in non-small-cell lung cancer: pulmonary function, prediction, and prevention. Int J Radiat Oncol Biol Phys (2005) 63:5–24 10.1016/j.ijrobp.2005.03.047 15963660

[42] Ghafoori P Marks LB Vujaskovic Z Kelsey CR Radiation-induced lung injury. Assessment, management, and prevention. Oncology (Williston Park) (2008) 22:37–47; discussion 52–33. 18251282

[43] Ghadjar P Zelefsky MJ Spratt DE Munck af Rosenschold P Oh JH Hunt M Impact of dose to the bladder trigone on long-term urinary function after high-dose intensity modulated radiation therapy for localized prostate cancer. Int J Radiat Oncol Biol Phys (2014) 88:339–44 10.1016/j.ijrobp.2013.10.042 24411606 PMC4581453

[44] Prosnitz RG Schneider L Manola J Rocha S Loffredo M Lopes L Tamsulosin palliates radiation-induced urethritis in patients with prostate cancer: results of a pilot study. Int J Radiat Oncol Biol Phys (1999) 45:563–6 10.1016/S0360-3016(99)00246-1 10524406

[45] Zelefsky MJ Ginor RX Fuks Z Leibel SA Efficacy of selective alpha-1 blocker therapy in the treatment of acute urinary symptoms during radiotherapy for localized prostate cancer. Int J Radiat Oncol Biol Phys (1999) 45:567–70 10.1016/S0360-3016(99)00232-1 10524407

[46] Merrick GS Butler WM Wallner KE Lief JH Galbreath RW Prophylactic versus therapeutic alpha-blockers after permanent prostate brachytherapy. Urology (2002) 60:650–5 10.1016/S0090-4295(02)01840-X 12385927

[47] Elshaikh MA Ulchaker JC Reddy CA Angermeier KW Klein EA Chehade N Prophylactic tamsulosin (Flomax) in patients undergoing prostate 125I brachytherapy for prostate carcinoma: final report of a double-blind placebo-controlled randomized study. Int J Radiat Oncol Biol Phys (2005) 62:164–9 10.1016/j.ijrobp.2004.09.036 15850917

[48] Symon Z Daignault S Symon R Dunn RL Sanda MG Sandler HM Measuring patients’ expectations regarding health-related quality-of-life outcomes associated with prostate cancer surgery or radiotherapy. Urology (2006) 68:1224–9 10.1016/j.urology.2006.08.1092 17141840

